# *Bacillus subtilis* G01: A Multifunctional Agent with Broad-Spectrum Antimicrobial Activity and Digestive Enzymes for Sustainable Agriculture and Animal Husbandry

**DOI:** 10.4014/jmb.2510.10049

**Published:** 2026-03-25

**Authors:** Yongen Yan, Jie Lin, Dongli Chen, Jiang Pi, Jun-Fa Xu, Xiaofang Zhong, Lingming Chen

**Affiliations:** 1 Institute of Laboratory Medicine, School of Medical Technology, Guangdong Medical University, Dongguan, 523808, P.R. China; 2 Marine Biomedical Research Institute, Guangdong Medical University, Zhanjiang, 524023, P.R. China; 3 Dongguan Key Laboratory of Drug Design and Formulation Technology, Biomedical Innovation Center, School of Pharmacy, Guangdong Medical University, Dongguan, 523808, P.R. China

**Keywords:** *Bacillus subtilis* G01, Biocontrol, Probiotic

## Abstract

*Bacillus subtilis* (*B. subtilis*), a versatile microorganism widely applied in agriculture and animal husbandry, has significant potential for supporting food security through crop protection and livestock productivity. On the basis of genomic and in vitro evidence, this study characterized a novel *B. subtilis* strain, G01, highlighting its potential as a candidate for biological control and food probiotics. Whole-genome sequencing revealed that G01 harbors nine secondary metabolite biosynthesis gene clusters, which play crucial roles in the biological control of microbial pathogens. Crucially, for potential food and feed-related uses, in vitro assays confirmed the potent broad-spectrum antimicrobial activity of G01 against phytopathogens and zoonotic bacteria, in addition to its ability to efficiently hydrolyze proteins and cellulose. Comparative genomics revealed unique gene clusters associated with antibacterial and probiotic-related functions in G01. Furthermore, genes involved in quorum sensing, biofilm formation, and spore production provide genomic support for its environmental resilience — a key characteristic for candidate biocontrol agents and probiotic precursors that target sustainable crop protection and livestock gut health management. This comprehensive genomic and *in vitro* functional analysis positions *B. subtilis* G01 as a promising candidate strain with theoretical potential for enhancing safety and efficiency in agricultural and livestock applications, laying a foundation for future in vivo validation of its practical efficacy.

## Introduction

*Bacillus*, one of the earliest bacteria found in humans, has been extensively researched as a biological control agent because of its strong stress resistance and environmental adaptability. As a representative strain, *Bacillus subtilis* is widely distributed in soil, rotting and other organic matter. *B. subtilis* can produce components, such as surfactin and fengycin, that can inhibit the growth of bacteria [1]. This study identified it as an ideal substitute for antibiotics. Moreover, Hinarejos *et al*. demonstrated that *B. subtilis* IAB/BS03 is an effective biocontrol agent that reduces the incidence of gray mold on tomato plants when it is sprayed on tomato foliage [2]. Consequently, over the last decade, *B. subtilis* has been extensively applied in the fields of biocontrol and the enhancement of plant growth.

*B. subtilis* is among the most commonly utilized probiotics. In recent years, the widespread use of antibiotics, despite providing considerable economic benefits to the livestock and poultry industry, has led to increasing concerns regarding bacterial resistance, superbugs, drug residues and ecological pollution [3]. Consequently, the search for a novel, safe and environmentally friendly substitute for antibiotics to maintain the intestinal health of livestock and improve animal productivity efficiency has become a compelling necessity. Probiotics have been proven to improve growth performance and reduce disease risk [4] and have the potential to serve as substitutes for traditional antibiotics [5, 6]. A previous study showed that *B. subtilis* can influence the gut microbiota of animals and maintain the microecological balance of the gut by depleting the oxygen in the gut and forming an environment favorable for the growth of anaerobic bacteria [7]. However, another study revealed that the addition of *B. subtilis* preparations to the diet significantly increased the number of intestinal probiotics, such as *lactobacilli*, while simultaneously reducing the count of harmful microbes, including *Escherichia coli* and *Salmonella* spp. [8]. Similarly, Wang *et al*. reported that adding *B. subtilis* to rations improved the growth performance of piglets [9]. These findings highlight the potential of *B. subtilis* as a probiotic agent in the field of animal feed production.

In the present study, we combined comprehensive bioinformatics, genomic comparative analyses and *in vitro* experimental methods to reveal the complete genome of *B. subtilis* G01, obtaining key information such as strain genomic characterization information, gene function annotation and classification, phylogenetic evolution and secondary metabolites, and revealing the antimicrobial activity and probiotic properties of *B. subtilis* G01. These results provide a solid theoretical foundation for the further development and application of *B. subtilis* G01 as a biocontrol and probiotic agent.

## Methods

### Bacterial Strains, Culture Media, and Growth Conditions

*Bacillus subtilis* strain G01 was obtained through isolation from air in a laboratory in Guangdong Province. The isolated *B. subtilis* G01 was placed into LB medium or LB broth (containing 10 g/l peptone, 5 g/l yeast extract, and 10 g/l NaCl, pH 7.2; 15 g/l agar was added to the plates when required) and cultured in a microaerobic atmosphere at 37°C for 20–24 h. To maintain the culture, the bacteria in the media were mixed with 30% glycerin and stored at -80°C. The GenBank login number of *B. subtilis* G01 is CP174155.

### DNA Extraction, Genome Sequencing and Assembly

The experimental operations were carried out following the standard procedures supplied by Oxford Nanopore Technologies (ONT) [10]. We extracted the genomic DNA of *B. subtilis* G01 via a bacterial DNA extraction kit (Omega Co.), following the manufacturer’s instructions. According to the manufacturer's protocol, DNA was extracted via Nanodrop (Thermo Fisher Scientific, USA), a Qubit 2.0 fluorometer (Life Technologies, USA) and 0.35% agarose gel electrophoresis for purity, concentration and integrity inspection. Subsequently, large fragments of DNA were recovered via the BluePippin fully automated nucleic acid recovery system, and library construction was performed via the SQK-LSK109 linking kit. Finally, the bacterial samples were sequenced via the Nanopore sequencing platform according to standard procedures. Canu v1.5 software uses default parameters to filter out joints, low-quality and short segments (lengths less than 2,000 bp), and 127,831 subreads were assembled, with an average length of 9,427 bp [11]. Pilon v1.22 software was used for further error correction with the second-generation data, and the genome with higher accuracy was obtained for subsequent analysis [12]. Next, unmapped reads were extracted from the comparison via SAMTOOLS, and plasmids were identified via SPADES [13].

### Functional Annotation

Gene prediction was performed via the software Prodigal v2.6.3, which uses dynamic programming algorithms to predict genes in newly sequenced genomes with high accuracy [14]. The Rfam database was combined with Infernal v1.1.3 software to identify three types of rRNA in the genome [15, 16]. The tRNAs in the genome were identified via tRNAscan-SE v2.0 software [17]. CRISPR prediction of the genome was performed via CRT v1.2 software [18]. IslandPath-DIMOB v0.2 software was used to predict gene islands in the bacterial genomes [19]. PhiSpy v2.3 software was used to identify prophage sequences in the bacterial genomes [20]. AntiSMASH v5.0.0 software was used to identify and analyze biosynthesis-related gene clusters in bacterial genome sequences [21]. To further analyze the G01 gene, we used the nonredundant protein (Nr) database created by NCBI, KEGG, eggNOG, SwissProt, and TrEMBL for BLAST annotation of the predicted G01 gene on the basis of sequence homology. Blast2GO v2.5 software was used for GO annotation on the basis of the GO database [22]. Protein family (Pfam) annotation was performed with the Pfam database [23]. Furthermore, the genome of strain G01 was annotated via the carbohydrate active enzymes (CAZy) database, transporter classification (TCDB) database, antibiotic resistance gene (CARD) database, pathogen–host interaction (PHI-base) database, and virulence factor (VFDB) database. Secretory proteins (proteins containing transmembrane helices were removed from proteins containing signal peptides) were identified via SignalP v4.0 software [24]. The genome circle map was generated via Circos v0.66 [25]. Genome-wide metabolic pathways were predicted via the RAST server [26]. On the basis of the whole-genome sequencing of *B. subtilis*, we used MEGA X and iTOL in combination with other genome sequencing results to construct an evolutionary phylogenetic tree [27, 28]. In this way, we can more accurately determine the kinship of *B. subtilis* strains and thus better guide the application of microorganisms. All analyses were performed with the default parameters.

### Antimicrobial Activity

We tested the antibacterial activity of the isolate via agar well diffusion assays. We adjusted the concentration of the indicator bacteria cultured in various media to 1 × 10^6^ CFU/ml and then added 1 ml of this bacterial suspension to 20 ml of 45°C nutrient agar medium. After mixing well, the mixture was poured into sterile petri plates containing sterile Oxford cups (Φ6 mm × 8 mm × 10 mm). After the agar solidified, sterile forceps were used to remove the Oxford cups. Then, 200 μl of bacterial supernatant, which was obtained from cultures grown at 37°C with shaking at 180 r/min for 24 h and centrifuged at 12,000 r/min for 5 min, was added to each well. One well received nutrient broth medium as a blank control. Once the supernatant had diffused into the agar, the plates were incubated at 37°C for 18–24 h. The diameter of the inhibition zones was then observed and measured.

### Probiotic Properties

The proteolytic activity of G01 was studied via agar well diffusion assays using skim milk plate media (5 g/l beef extract, 10 g/l peptone, 1 g/l NaCl, and 16 g/l agar, pH 7.2). Single colonies of G01 were inoculated onto this medium and incubated at 37°C for 24–36 h. Protease activity was assessed by measuring the diameter of the degradation zones formed.

Similarly, the cellulase hydrolysis activity of G01 was studied via an agar well diffusion assay via carboxymethyl cellulose sodium-congo red medium (1.5 g/l disodium hydrogen phosphate, 1.5 g/l ammonium sulfate, 0.3 g/l magnesium sulfate · 7H_2_O, 0.2 g/l calcium chloride · water, 5 g/l sodium chloride, 0.3 g/l urea, 10 g/l carboxymethyl cellulose sodium, 0.3 g/l peptone, and 16 g/l agar, pH 7.2). Colonies were inoculated onto the media via an inoculation loop and then incubated at 37°C for 24–36 h. The cellulase activity was assessed by measuring the diameter of the degraded zones.

In addition, we quantitatively assessed the ability of the strains to form biofilms via crystal violet staining. Briefly, the bacteria that had been cultured overnight were diluted to 1 × 10^6^ CFU/ml. Subsequently, 200 μl of this diluted bacterial mixture was added to each well of a 96-well plate (with six replicate wells), and the mixture was incubated at 37°C for 24 h, with blank medium used as a control. After incubation, the medium was removed, and the cells were washed three times with PBS to remove impurities and bacteria. Then, 200 μl of methanol was added to each well to fix the biofilms. After 15 min, the methanol was removed, and the wells were dried. Next, 0.1% crystal violet was added to each well, and the mixture was stained for 5 min. The excess staining solution was removed, and then 200 μl of 33% glacial acetic acid was added to each well. The plates were shaken vigorously on a microplate reader for 20 min, and the optical density was measured at a wavelength of 595 nm.

### Comparative Genomic Analysis and Substitution Rate Estimation

DNA comparison and phylogenetic tree construction using the *gyrB* gene from 98 whole-genome sequenced *B. subtilis* plants [29]. The maximum likelihood method was used. The tree was tested for reliability through 1000 repeated bootstrap methods. The two strains closest to *B. subtilis* G01 were selected for comparative genomic analysis. Collinearity analysis of G01 and two other strains was performed via TBtools software, and a synteny map with default parameters was generated [30]. The OrthoVenn3 website was used to compare and annotate orthologous gene clusters of different strains [31]. The comparison adopts the default parameters of the website, that is, the OrthoMCL algorithm, with an inflation value of 1.5 and an E value of 1e-2. OrthoVenn3 is an online tool for comparing and analyzing proteomic data from multiple species. It uses visualizations to help researchers identify common and specific sets of proteins that reveal functional and evolutionary relationships between different species. To define an orthologous gene cluster, we use whole-to-all sequence alignment and then use the Markov cluster algorithm (MCL) for clustering analysis so that genes with similar sequence characteristics are aggregated together to form orthologous gene clusters. AmiGO2 (https://geneontology) was subsequently used for GO annotation of orthologous gene clusters.

The nonsynonymous replacement rate (Ka) and synonymous replacement rate (Ks) of each homologous gene were subsequently calculated, and the ratio of the two (Ka/Ks) was used to measure the rate of genome evolution in the G01 genome. Orthologous genes with a Ka/Ks > 0.5 or 1 were considered genes under positive selection; orthologous genes with 0.1 < Ka/Ks < 0.5 were considered affected by weak positive selection; and orthologous genes with a Ka/Ks < 0.1 were considered affected by negative selection. This Ka/Ks ratio has proven useful for identifying genes under positive selection according to previous reports [32].

## Results

### Genome Sequencing and Assembly

**Fig. 1:**
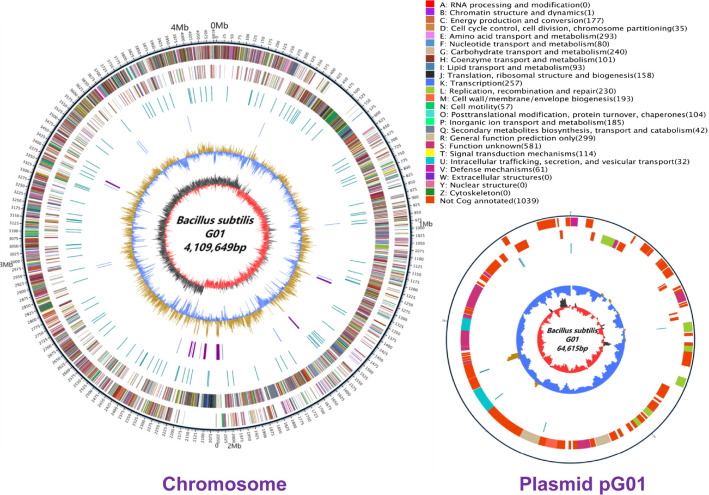
Whole genome of *B. subtilis* G01. The outermost circle marks the size of the genome, and each scale is 5 kb. The second circle and the third circle are the genes on the positive and negative chains of the genome, respectively, and different colors represent different functional classifications of the COG. The fourth circle represents repeated sequences. The fifth circle represents tRNA and rRNA, the blue circle represents tRNA, and the purple circle represents rRNA. The sixth circle represents the GC content, and the light yellow part indicates that the GC content of this region is higher than the average GC content of the genome; the higher the peak value is, the greater the difference between the average GC content and the blue part indicates that the GC content of this region is lower than the average GC content of the genome. The innermost ring is GC-skew, with dark gray representing regions with more G than C and red representing regions with more C than G. CDSs are colored according to the main COG functional classification categories.

The genome of *B. subtilis* G01 was sequenced via nanopore sequencing to obtain complete genomic information. The genome of the G01 bacterium comprises a circular chromosome of 4,109,649 bp in length and a circular plasmid of 64,615 bp in length (Fig. 1). The basic features of the *B. subtilis* G01 genome can be found in Table 1. Chromosomes make up 43.40% of the total GC content and harbor 4285 predicted genes. The plasmid has a GC content of 38.00% and contains 87 predicted genes. On the chromosome, we also detected 88 tRNA genes, 30 rRNA genes, and 11 clustered regularly interspaced short palindromic repeats (CRISPR), along with a total of three prophage regions, but these regions were not found in the plasmid genome. Moreover, nineteen genomic islands were found in the chromosome, and one genomic island was found in the plasmid.

### Functional Annotation

Cluster analysis of orthologous protein groups (COGs) revealed 1039 genes with no annotation and 581 genes with unknown functions. The remaining genes were inferred to possess functional properties, and the leading ten COG functional categories included “general function prediction only,” “amino acid transport and metabolism,” “transcription,” “carbohydrate transport and metabolism,” “replication, recombination and repair,” “cell wall, membrane, envelope biogenesis,” “inorganic ion transport and metabolism,” “energy production and conversion,” “translation, ribosomal structure and biogenesis,” and “signal transduction mechanisms” (Fig. 1). In the pG01 plasmid genome, we identified a total of 87 genes. Except for 17 functionally labeled genes, the functions of most of the genes encoded by the pG01 plasmid are unknown. However, we surprisingly found that GE004286 (VanZ family protein) has low levels of resistance to teicoplanin, an antibiotic used to treat gram-positive bacterial infections [33]. GE004327 (Repressor Rok) has additionally been found to suppress no fewer than 20 genes responsible for encoding proteins related to membrane localization and secretion, including some that code for products with antibiotic activity [34] (Table S1).

**Fig. 2:**
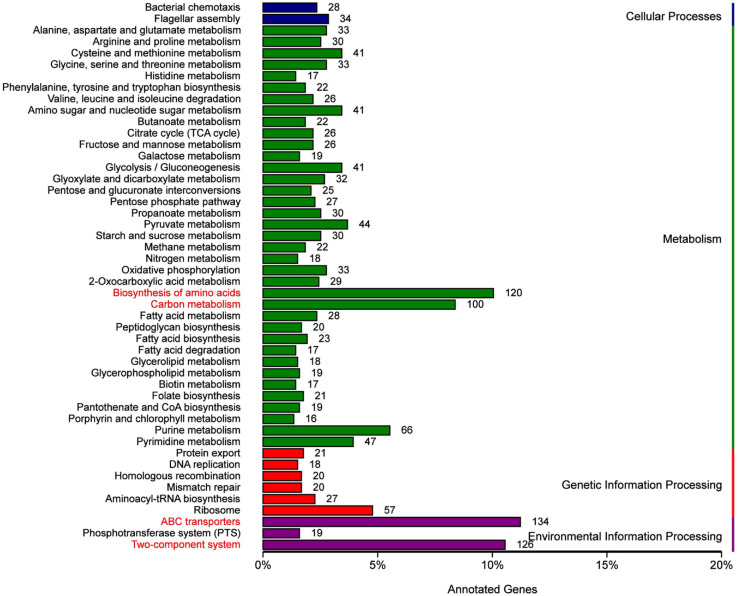
The results of classification statistics of KEGG pathway annotation.

**Fig. 3:**
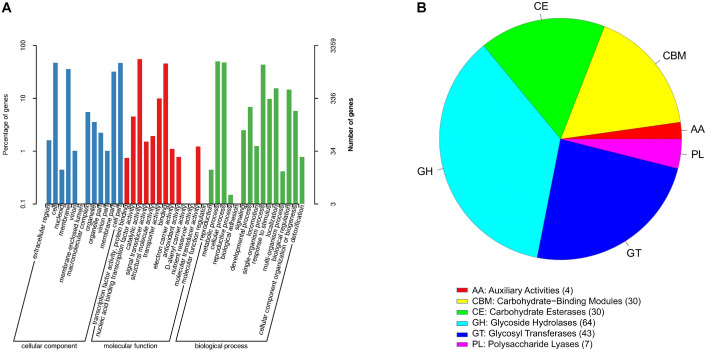
Functional annotation of *B. subtilis* G01. (**A**) GO function annotation categorical statistics. (**B**) Carbohydrate-active enzyme distribution scale map.

Next, we conducted enrichment analyses via Gene Ontology (GO) and Kyoto Encyclopedia of Genes and Genomes (KEGG) enrichment analyses to achieve functional annotation of candidate genes and assess the gene ontology (GO) and functional classification of these predicted genes. Kyoto Encyclopedia of Genes and Genomes (KEGG) pathway analysis revealed that the genes predicted for G01 were highly enriched in the ABC transporter and two-component system pathways, with the pathways for amino acid biosynthesis and carbon metabolism being next (Fig. 2). In addition, three categories of GO terms were enriched: cellular components, molecular functions, and biological processes. The significantly enriched terms in the cellular component category included “cell”, “membrane”, “cell part”, and “membrane part”. Those in molecular functions that were notably enriched included catalytic activity, binding activity, and transporter activity. For biological processes, the significantly enriched terms covered metabolic processes, cellular processes, single-organism processes, biological processes, and responses to stimuli. (Fig. 3A). In addition, VFDB identified a total of 722 genes, most of which were associated with virulence factors such as “capsule,” “LPS,” and “flagella” (Fig. S2). According to the annotation results from the PHI database (Fig. S3), 1,286 genes involved in bacterial‒host interactions were classified into six categories. The most active category was “reduced virulence” (64%), followed by “unaffected pathogenicity” (20%) and “loss of pathogenicity” (5%).

### Carbohydrate-Active Enzymes (CAZymes)

CAZymes represent a category of enzymes associated with sugar synthesis, breakdown, and modification, and they serve key functions in breaking down sugars into their constituent parts [35]. A total of 178 genes encoding carbohydrate-active enzymes (CAZymes) were identified in the G01 genome sequence, with glycoside hydrolases (GHs) and glycosyltransferases (GTs) dominating the repertoire, followed by carbohydrate esterases (CEs), carbohydrate-binding modules (CBMs), polysaccharide lyases (PLs), and auxiliary activities (AAs) (Fig. 3B). Among these, GHs refers to a general category of enzymes capable of acting on various glycosides or oligosaccharides to catalyze the hydrolysis of glycosidic bonds, especially those that act on and hydrolyze the glycosidic bonds of carbohydrates. GTs, on the other hand, constitute an important class of naturally occurring glycosidic bond-synthesizing enzymes that are increasingly used in the synthesis of complex sugars and glycoconjugates [36].

### Secondary Metabolism Gene Clusters and Biocontrol Function

In recent years, *B. subtilis* has become a research hotspot for biocontrol agents because *B. subtilis* inhibits the growth of pathogenic microorganisms such as fungi and bacteria [37]. With increasing research on *B. subtilis*, we have succeeded in identifying a number of *B. subtilis* isolates with large clusters of biosynthetic genes that encode gene products for the production of secondary metabolites [38]. Moreover, these synthesized gene clusters can also provide *B. subtilis* with more abundant nutrients, thus effectively promoting its growth and reproduction. By sequencing the whole genome of *B. subtilis* G01, we identified nine gene clusters related to secondary metabolite biosynthesis, which play important roles in controlling the biocontrol of microbial pathogens (Table 2). On the basis of the labeled genes, *B. subtilis* G01 showed high genetic competence in the synthesis of type III polyketide synthases (T3PKS), nonribosomal peptide synthases (NRPS), terpenes, betalactones, ribosomally synthesized and posttranslationally modified peptides (RiPP-like) and sactipeptides. Two gene clusters encode antifungal metabolite enzymes (*e.g.*, surfactin and fengycin), two gene clusters encode antimicrobial metabolite enzymes (*e.g.*, bacilysin and subtilosin A), and one gene cluster encodes metabolite synthetases involved in nutrient uptake (*e.g.*, bacillibactin). In addition, the enzyme functions encoded by the other four gene clusters were not annotated, indicating that they are strain-specific gene clusters for G01. Among these genes, two terpene gene clusters, GE000206--GE000225 and GE001156--GE001177, were identified. Terpenes and their derivatives have been shown to have strong antibacterial activity and have great potential for antimicrobial resistance, so they are expected to be potential alternatives to overcome antibiotic resistance [39]. This antimicrobial property gives G01 better biological control capabilities.

Moreover, *B. subtilis* G01 showed antagonistic activity against gram-positive bacteria such as *Staphylococcus aureus* and *Micrococcus luteus* and fungi such as *Candida albicans* and *Aspergillus niger* but also showed enhanced antagonistic activity against some gram-negative bacteria, such as *Ralstonia solanacearum* and *Legionella pneumophila*. However, it also showed no antimicrobial activity against several gram-negative bacteria, such as *E. coli*, *Pseudomonas aeruginosa* and *Listeria monocytogenes* (Table 3). In conclusion, the present study revealed the important role of *B. subtilis* G01 in biocontrol, and by characterizing its biosynthesis-related gene cluster, we identified many genes related to the synthesis of antimicrobial compounds, which encode mainly enzymes for the synthesis of a wide range of antimicrobial compounds. However, since the use of G01 requires further investigation, we also need to further explore its biocontrol function.

### Antibiotic Resistance

Antibiotic resistance refers to the enhanced tolerance of bacteria to antibiotics or the phenomenon whereby bacteria acquire resistance to these agents, and this represents a common inherent trait of the genus Bacillus. The reason for this resistance is that it involves a variety of antibiotic resistance genes, which can be spread between different strains through horizontal gene transfer (HGT) and other methods, leading to the rapid spread of antibiotic resistance [40, 41]. Therefore, the study of antibiotic resistance is highly important in the field of medicine, especially in the prevention and treatment of diseases caused by bacteria.

Here, we identified 11 antibiotic resistance genes involved in antibiotic resistance: efflux pumps (ykkd, ykkc, blt, and bmr); the ATP-binding membrane protein tmrB; the ABC-F ATPase ribosome protection protein ymlR; and aadK, a chromosome encoding the aminoglycoside nucleotide transferase gene in *B. subtilis*; the chromosome macrolide phosphotransferase mphK from *B. subtilis*; the macrolide-lincosamide-streptogramin B efflux pump (lmrB); the membrane protein mprF, which modifies negatively charged phosphatidylglycerol on the membrane surface and provides resistance to cationic peptides that damage cell membranes; and a point mutation targeting the *pgsA* gene of *B. subtilis* (Table S4). These 11 antibiotic resistance genes can be resistant to a variety of antibiotics (such as daptomycin, pleuromethine, lincoamide, streptomycin A, itamycin, lincomycin, and streptomycin) to protect themselves from antibiotics produced by other microorganisms and improve survival [42-46].

### Prophage Elements

Phages are an important component of many bacteria. They are small, independently replicating bacterial viruses. They can also integrate into their genomes as phages and replicate passively with their hosts [47]. Phages were found in approximately half of the bacterial isolates [48]. They are usually found in complex communities, including the mouse [49] and human gut [50]. Therefore, the correct identification of phages is key to understanding the bacterial genome and revealing its genetic potential [51].

Within the G01 bacterial strain, three phage regions were identified (Fig. S2). All three prophage regions were located on the chromosome, and no prophage regions were detected on the plasmid. The prophage 1 region was located between 257,644 and 402,188 bp in length, with a length of 144,545 bp, and consisted of 164 coding sequences (CDSs) consisting of glutamyltransferase, penicillin-binding protein, nonribosomal peptide synthase, enoyl-coenzyme A hydratase, metallothioltransferase, and autolysin. The prophage 2 region is located between 855,187 and 939,975 bp with a length of 84,789 bp and consists of the phage-like components PBSX protein XkdP, positive control sigma-like factor, ISLmo1-like transposase, mannuronic acid dehydratase, zinc-binding alcohol dehydrogenase, uroporphyrinogen-III synthase and 98 other CDSs. The prophage 3 region is located between 1,644,830 and 1,683,768 bp with a length of 38,939 bp and consists of PLP-dependent aminotransferase family proteins, AraC family transcriptional regulators, glutamine amidotransferases, phosphatases, endopeptidases, membrane proteins, and 42 other CDSs.

### Secreted Proteins Involved in Host–Bacteria Interactions

In host-associated bacteria, surface and secreted proteins are primarily used to mediate nutrient acquisition and interactions with host cells, thereby influencing host tissue localization [52]. They play an important role in host-associated bacteria, and they also play a vital role in the life of bacteria. Quorum sensing regulates gene expression by responding to fluctuations in cell population density and is a way for bacteria to communicate and coordinate their behavior with other bacteria by releasing signaling molecules [53, 54]. *B. subtilis* can sense changes in the surrounding environment by releasing signaling molecules and regulating its growth and metabolism. Therefore, the quorum sensing ability of *B. subtilis* is highly important for its survival and competition in soil [55]. The genome of *B. subtilis* G01 contains genes encoding host‒bacterial interaction proteins that are secreted by bacterial cells or involved in quorum sensing (Table S5).

Among these proteins secreted by *B. subtilis*, we surprisingly found that ABC transporter substrate-binding proteins accounted for a considerable portion. ATP-binding cassette (ABC) transporter proteins are the major transporter proteins that couple energy stored in adenosine triphosphate (ATP) to move molecules across membranes; they mediate the uptake of essential nutrients and the transport of molecules in the cell and are also closely related to multidrug resistance in bacteria and eukaryotic cells [56]. Among the ABC transporter substrate-binding proteins secreted by G01, GE002001 and GE003147 encode glycine betaine ABC transporter substrate-binding proteins and the osmoprotectant agent ABC transporter substrate-binding proteins, which can increase the growth rate of *B. subtilis* in high osmotic pressure media. It is also allowed to proliferate under conditions that strongly inhibit its growth [57]. The genes GE001517, GE002976 and GE003074 encode polysaccharide lyase, pectin lyase and glycoside hydrolase, respectively, possibly because G01 has a better ability to digest food and thus obtain more nutrients [58]. The genes GE001873 and GE002136 encode penicillin-binding proteins, which can increase the resistance of G01 to antibiotics and inhibit the growth of some plant pathogens, increasing its antibacterial and biological control functions [59]. The genes GE000203, GE000826, GE001307 and GE001312 secrete proteins associated with cell wall hydrolysis to help bacteria absorb nutrients and adapt to different growth environments [60]. Some genes are involved in spore formation (GE000718, GE001333, GE002153, and GE003775), which is necessary for bacteria to resist external stress and spread [61]. In addition, the genes GE002136 and GE002567, encoding the penicillin-binding protein PBP4B and polysaccharide deacetylase, also have spore formation functions [62, 63]. In *B. subtilis* G01, the quorum-sensing peptide ComX, which has a positive effect on spore formation [64], was identified. Moreover, the Rap-Phr system was also identified in the G01 genome, which plays an important role in the formation of G01 biofilms *in vitro* and in plant root colonization [65].

### Probiotic Properties

In recent years, many studies have shown that *B. subtilis* can inhibit the growth of harmful bacteria. By producing antibiotics and other inhibitory substances, *B. subtilis* can help maintain the balance of the intestinal flora of animals and reduce the growth of harmful bacteria, thus reducing the occurrence of intestinal infections in animals. Moreover, *B. subtilis* is also able to promote the absorption of nutrients in animals. It can decompose cellulose and other indigestible substances in food, help improve the utilization of nutrients by animals, and promote the growth and development of animals. Therefore, *B. subtilis* is now widely used as a probiotic in animal feed. According to experiments on broilers, white shrimp and fish, the addition of several *B. subtilis* strains as probiotics to feed can significantly improve the growth performance of animals, suggesting that *B. subtilis* can be a safe and beneficial animal probiotic preparation [66, 67, 68]. In a human trial, *B. subtilis* was also shown to be safe and suitable for use as a probiotic [69]. As probiotics are used in animal feed and nutrition, their antibacterial properties against pathogenic bacteria are particularly important [70]. *B. subtilis* G01 has a gene cluster that synthesizes secondary metabolites with strong antimicrobial activity (Table 2). In addition, G01 shows significant antibacterial activity in both antibacterial and antifungal aspects (Table 3). Furthermore, we found that G01 can effectively hydrolyze protein and cellulose (Table 4), indicating that it is a good raw material for protease and cellulose. This effective hydrolysis function helps animals digest food better and thus obtain more nutrients. Biofilm formation is the key to colonization [71]. Our study revealed that G01 can form strong adhesion biofilms (Table 4), which play important roles in adhesion and colonization. Therefore, our findings provide important data supporting the use of *B. subtilis* G01 as a probiotic.

### Comparative Genomic Analysis

Phylogenetics is the basis of genome analysis and bioinformatics analysis of microorganisms. It uses a conserved nucleotide sequence on a gene to reconstruct the evolutionary relationship of similarity between microorganisms to determine the relationship between different microorganisms [72]. Phylogenetic trees can be used to reveal the evolutionary relationships between species and assist in biological classification and identification, which is highly important for bioinformatics research and application. 16S rRNA is a part of the ribosomal RNA of bacteria and archaea, and its sequence varies among different species. By comparing the 16S rRNA sequences of different organisms and constructing a phylogenetic tree, the evolutionary relationships between them can be revealed, thus helping us to understand the evolutionary history and origin of organisms [73]. However, although the 16S rRNA sequence is a commonly used molecular marker, it also has certain limitations, mainly in the differentiation of closely related taxa, which may affect its accuracy in the construction of phylogenetic trees. Over time, an increasing number of people are interested in the use of alternative loci, such as *gyrA* and *gyrB*, as molecular timekeepers [29]. This approach could not only help people identify the differences between different species more accurately but also help us better understand the evolutionary relationships between species. Variations in the *gyrB* gene sequence differ between *B. subtilis* populations [74, 75, 76]. In general, 16S rRNA sequences are employed to characterize evolutionary relationships among distantly related taxa, whereas *gyrB* sequences are better suited for deducing relationships within and between genera.

**Fig. 4:**
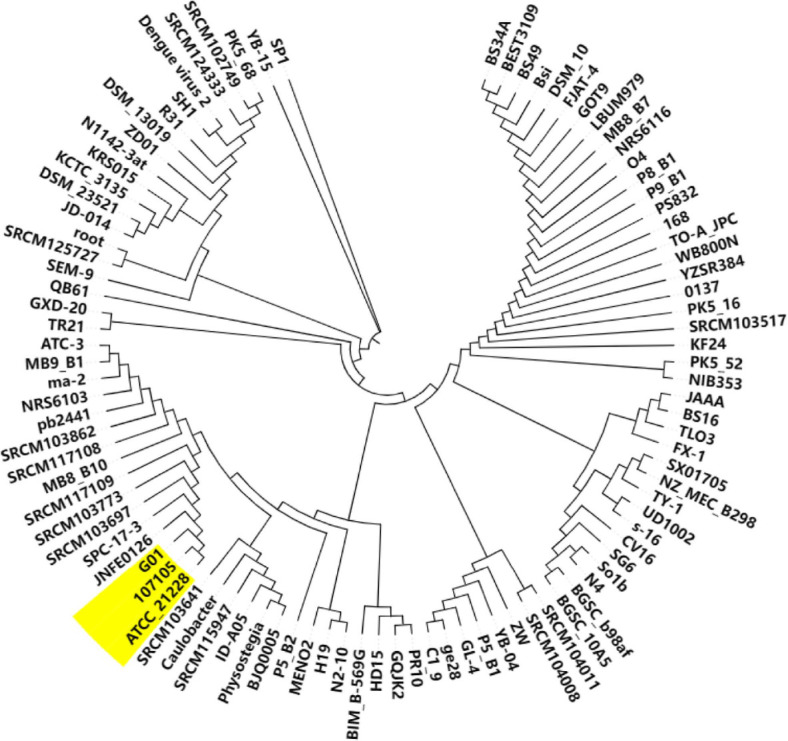
Maximum-likelihood tree of *B. subtilis* G01 based on *gyrB* nucleotide sequences. This analysis involved 98 nucleotide sequences from all complete genome-sequenced *B. subtilis* strains via the maximum likelihood method. The bold branches indicate the strains closest to the G01 strain.

**Fig. 5:**
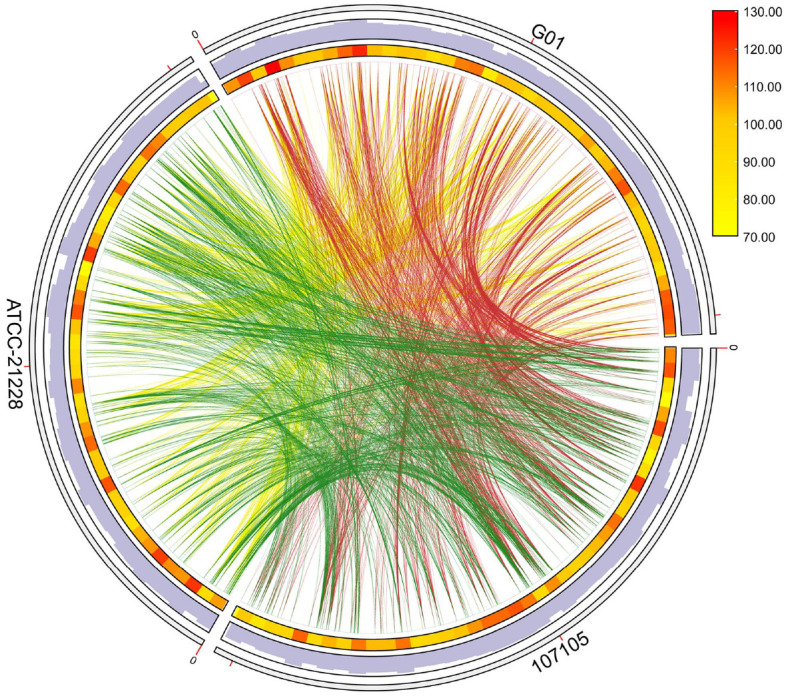
Synteny map of *B. subtilis* strains G01, 107105, and ATCC_21228 genomes. Genomic comparison of the *B. subtilis* G01 strain against closely related representative *B. subtilis* strains 107105 and ATCC_21228. The outermost circle is labeled with the genome name; the second and third circles are gene frequencies; the inner regions with different colored lines indicate collinearity between genomes.

To assess the overall genetic diversity of G01 compared with that of other sequenced strains of *B. subtilis*, we constructed a maximum likelihood tree for G01 on the basis of the *gyrB* nucleotide sequence via MEGA X [27]. The genome of strain G01 was compared with all fully sequenced *B. subtilis* genomes in the GenBank database (Fig. 4). Compared with selecting distantly related strains or randomly increasing the number of reference strains, closely related strains have much smaller genetic differences. This allows for the elimination of nonspecific interference caused by long-term species divergence, enabling more accurate identification of the gene clusters, functional variations, and evolutionary adaptations unique to G01. We found that G01 was closely related to two subtaxa of this genus, *B. subtilis* 107105 and ATCC_21228. On this basis, a comparative genomics study was carried out on the genomes of the three strains via TB tools software. Using *B. subtilis* G01 as a reference genome, the co-origin region between G01 and two other representative sequenced strains, *B. subtilis* 107105 and ATCC_21228, was identified (Fig. 5). The two pairs of data, G01 and 107105, as well as G01 and ATCC_21228, contain 1,135 and 1,188 collinear blocks, respectively. The results revealed that the G01 genome was more closely related to the ATCC_21228 genome than to the 107105 genomes.

In addition, both G01 and ATCC_21228 contain a single plasmid, pG01 (encoding 87 genes), and pLDW-15 (encoding 111 genes). A comparison of the full-length nucleotides of the two plasmids revealed that both contained genes encoding the UV damage repair protein UvrX and genes encoding site-specific integrases. Among them, UvrX is expected to be a DNA polymerase that contributes to the UV stress response system [77], whereas site-specific integrase is an enzyme that specifically catalyzes the insertion or deletion of DNA fragments on plasmids or chimeric DNA, playing an important role in bacterial genome rearrangement and gene expression regulation. The presence of both proteins helps bacteria adapt to changes in the environment and has an important effect on the growth and survival of host bacteria. In the G01 and ATCC_21228 genomes, with the exception of the above two genes encoding the same protein, the other genes were different, indicating that these different genes endow plasmids with specific functions. Two genes contained in pG01, GE004325 (encoding a single-stranded DNA-binding protein) and GE004327 (encoding Rok, a ComK repressor), are involved in spore formation [78, 79].

**Fig. 6:**
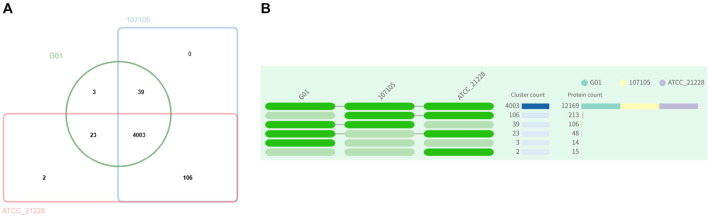
Comparison and annotation of orthologous gene clusters among *B. subtilis* strains G01, 107105, and ATCC_21228. Venn diagram showing the shared/unique orthologous gene cluster numbers of strain G01 with strains 107105 and ATCC_21228. A dark green cell indicates the presence of a cluster group in the corresponding species, and a light green bar represents the absence of a cluster group in that species.

To gain insight into gene and genome evolution, we carried out a comparative study on genome-wide homologous clusters of three strains, G01, 107105 and ATCC_21228, aimed at identifying homologous genes and gauging their similarity level. In total, 4003 core genome homologs were found (Fig. 6A). Compared with 107105 and ATCC_21228, G01 has a total of 3 specific gene clusters, including 14 genes (Fig. 6B). GO annotation of the specific gene clusters of these strains revealed that cluster 4012 contained an integral component of the membrane (GO:0016021), whereas the annotation results of the other two gene clusters were unknown (Fig. S3).

In addition, 221 singletons were found in the G01 strain. They are gene or protein sequences that occur in only one species. In other words, these gene or protein sequences were unique to the entire dataset and were not matched with sequences from other species. These individually existing sequences may have specific functions or characteristics and therefore have some importance when the genomes of different species are compared. The representative singletons are shown in Table S5. Among these, GE000156 and GE002358 encode the spore maturation protein CgeA and the spore coat protein CotF, which are associated with the occurrence and formation of bacterial spores [80, 81]. GE000255 and GE000256 encode the putative UV damage repair protein UvrX, which helps strains adapt to various extreme environments [82]. GE000308 encodes the palpastatin synthase subunit, an antifungal antibiotic and one of the most important nonribosomal lipopeptides produced by *B. subtilis*. Plipastatin has strong fungal toxic activity and is involved in inhibiting phospholipase A2 and biofilm formation [83]. Moreover, GE003915 encodes a peptidoglycan O-acetyltransferase, which also endows G01 with good antibacterial activity [84]. GE001993 encodes L-lactate permease, and the uptake and utilization of L-lactate are important for the formation of biofilms in *B. subtilis* [85]. The antitoxin YqcF, encoded by GE003983, is an antitoxin protein commonly used to neutralize the effects of toxins [86]. This antitoxin may play an important role in the bacterial immune system, helping bacteria fight off external toxin attacks. In general, the specific genes of G01 mentioned above have improved antibacterial, antitoxin and environmental adaptability.

The ratio of the nonsynonymous substitution rate (Ka) to the synonymous substitution rate (Ks) is widely used as an indicator of selection pressure for different species at the sequence level [87]. In molecular evolutionary analysis, Ka/Ks > 1 is generally considered a clear sign of positive selection, indicating that amino acid changes are beneficial for adaptation; Ka/Ks ≈ 1 suggests neutral evolution, whereas Ka/Ks < 1 indicates purifying selection, indicating that the function of the protein is constrained. To conduct a more detailed analysis, this study adopted a set of stricter thresholds, drawing on the classification strategies used in similar studies by Li *et al*.: orthologous genes with a Ka/Ks > 0.5 or 1 were considered genes under positive selection; orthologous genes with 0.1 < Ka/Ks < 0.5 were considered affected by weak positive selection; and orthologous genes with a Ka/Ks < 0.1 were considered affected by negative selection [32]. This classification helps to identify genes that, although not meeting the Ka/Ks > 1 criterion, still exhibit clear trends of nonneutral evolution. To assess the evolutionary pressure on homologous genes between G01 and its closely related strains, we used the TBtools Ka/Ks calculator to calculate the Ka/Ks ratios for the G01, 107105, and ATCC_21228 strains. The results of this calculation can reflect the interrelationships between genes, so a calculator is used to better understand the evolutionary dynamics between various strains. In addition, we performed a statistical analysis of the calculated results to determine which genes were important for the evolution of G01. The results revealed that among 4026 pairs of homologous genes between G01 and ATCC_21228, 1 pair had Ka/Ks values ranging from 0.5 to 1.0 (positive selection), and 6 pairs had Ka/Ks values ranging from 0.1 to 0.5 (weak positive selection). The same result was found for 4042 pairs of homologous genes between G01 and 107105 (Table 5). The genes associated with the evolution of G01 mainly encode “surfactin nonribosomal peptide synthetase SrfAA”, “surfactin nonribosomal peptide synthetase SrfAB” and “nonribosomal peptide synthetase DhbF”. Among these genes, surfactin nonribosomal peptide synthetase SrfAA and surfactin nonribosomal peptide synthetase SrfAB are involved primarily in the synthesis of surfactants (antibiotics synthesized by bacterial nonribosomal peptide synthase). Surfacins have been reported to have significant inhibitory effects on pathogens such as fungi, bacteria, viruses and Mycoplasma [88-92]. In addition, surfactants play important roles in biofilm formation and the movement and colonization of plant tissues and are involved in inducing plant resistance to pathogens [93-95]. These findings suggest that G01 may have good biological control potential. The nonribosomal peptide synthetase DhbF is a ferrisomal protein that plays an important role in bacteria [96]. DhbF is able to bind iron ions and transport them inside bacterial cells, thereby helping bacteria survive and reproduce in iron-deficient environments. However, it is necessary to approach this interpretation with caution. First, the number of genes subject to such selection pressure is extremely small, and their statistical significance and biological relevance need to be verified in larger collections of strains. Second, lower Ka/Ks values may also reflect the strong functional constraints inherent in these genes, meaning that the selective pressure underlying their purification is relatively low, rather than indicating significant adaptive evolution. Finally, the accuracy of Ka/Ks values derived from comparisons of limited closely related strains may be influenced by the quality of sequence alignment, the choice of evolutionary models, and sampling biases. Therefore, equating the Ka/Ks values of these genes directly with evidence that G01 possesses special evolutionary advantages in biological control or probiotic functions is still preliminary. While these genes may play important roles in the metabolic functions of G01, the causal evolutionary relationships between them and the unique phenotypes of the strain require further clarification through broader population genetic analyses, gene expression data, and functional experiments.

## Discussion

This study systematically characterized *B. subtilis* G01 through the integration of whole-genome sequencing, comparative genomics, and *in vitro* functional experiments. While extensive reports have documented the identification of *B. subtilis* strains for biocontrol or probiotic applications via whole-genome sequencing, most of these strains in agricultural and livestock applications have been limited to single-function utilization (Table S6). For example, the porcine-derived strain BS21 is primarily used as an antibiotic alternative probiotic to maintain the balance of the animal gut microbiota [1]; the rhizosphere isolate Bbv57 and the soil isolate KC14-1 focus on single biological control functions, specifically targeting plant soil-borne pathogens and broad-spectrum fungi, respectively [38, 97]; isolates from the gut of *Periplaneta americana* MC4-2, which control plant pathogens such as the tobacco black shank disease pathogen [98]; and DG101, which is isolated from Japanese natto, has been elucidated as a novel spore-forming probiotic strain with significant potential for human consumption [99]. However, the core value of this study lies in revealing a unique combination of genomic characteristics and phenotypic functions possessed by G01, which distinguishes it from common probiotic or biocontrol strains that typically have relatively single functions. Specifically, the novelty of G01 lies in its potential as a multifunctional agent that possesses both “targeted biocontrol” and “digestive-enhancing probiotic” properties.

In terms of antibacterial properties, G01 has a selective inhibitory spectrum that makes it particularly suitable for specific applications. Unlike typical probiotic strains that mainly target animal intestinal pathogens or biocontrol strains that specifically inhibit plant soil-borne fungi, G01 shows significant activity against important plant pathogenic bacteria such as *Pseudomonas aeruginosa*, as well as zoonotic pathogens such as *Legionella pneumophila*. In contrast, its inhibitory effect on common members of the animal intestinal flora is relatively weak (Table 3). This selectivity suggests that when used in livestock and poultry farming, G01 may be more effective in preventing and controlling specific pathogens introduced from the environment or feed rather than broadly disrupting the intestinal microbiota. This provides a unique approach for utilizing G01 as a “targeted” probiotic. With respect to its prebiotic effects, *in vitro* experiments confirmed that G01 possesses high levels of protease and cellulase activity (Table 4). Its ability to degrade both proteins and cellulose simultaneously means that when used as a feed additive, it can directly help animals improve their utilization of complex nutrients in their diet.

Successful colonization is a prerequisite for the long-term effectiveness of probiotics and biocontrol agents. This study confirmed that G01 possesses strong biofilm-forming ability (Table 4), which is a key trait enabling it to establish and maintain a population advantage on the host surface or in complex environments. Genomic analysis has provided molecular evidence to support this: G01 encodes a complete quorum sensing system, including genes such as ComX and Rap-Phr, as well as many genes related to the synthesis of extracellular polysaccharides. Together, these components constitute the genetic basis for its adhesion and aggregation. In addition, proteomic analysis has revealed several proteins that may be involved in host–environment interactions, such as peptidoglycan hydrolases, quorum-sensing signaling molecules, and potential adhesion factors. These proteins are likely to play a role in the initial attachment of G01 to plant roots or animal intestinal epithelium, in the acquisition of nutrients, and in its adaptation to the microenvironment.

HGT is a key mechanism by which bacteria acquire and spread antibiotic resistance. Here, we conducted a systematic assessment of the potential for HGT of the 11 antibiotic resistance genes present in *B. subtilis* G01. Specifically, ten of these genes (ykkd, ykkc, bmr, tmrB, ymlR, aadK, mphK, lmrB, mprF, and *pgsA*) are located in conserved chromosomal regions and are not associated with mobile genetic elements such as prophages, plasmids, or gene islands. Notably, the efflux pump gene blt is the only resistance gene located in the genomic island. However, the genes on this island form a functionally coordinated metabolic and detoxification module, including genes related to amino acid transport, heavy metal excretion, and redox metabolism. This finding suggests that the genomic island may have arisen through the integration of exogenous genetic material. Its core functions are related to nutrient transport, ion homeostasis, and metabolic regulation. The results indicate that, on the basis of the current genomic data, these genes do not pose a significant risk of spread. They represent resistance mechanisms specific to the G01 strain and therefore do not constitute a biosafety threat. Moreover, these genomic-based predictions require further validation through future in vitro and in vivo experiments.

From the perspective of potential advantages, some genes encoded by prophages may increase the environmental adaptability of G01: glutamyltransferase may be involved in nutrient utilization or stress detoxification [100], PLP-dependent aminotransferase may contribute to metabolic balance in complex ecological niches [101], and these functions may indirectly increase its biocontrol efficiency and probiotic properties. However, from the perspective of biosafety risks, the primary threat posed by prophages lies in their inducibility and their ability to mediate HGT. Stressors in the environment, such as ultraviolet radiation, exposure to antibiotics, or nutritional deficiencies, can trigger the lytic cycle of prophages, resulting in the release of progeny phages [102]. If these progeny phages carry functional genes that are transferred to other microorganisms through transduction, this could lead to the widespread dissemination of harmful traits, such as antibiotic resistance genes or virulence genes, within microbial populations. Consequently, whether these prophage elements affect the diversity of beneficial traits of G01 and the safe boundaries of their practical application still require further verification through targeted experiments.

The genomic basis that underlies these phenotypes also exhibits a degree of integration and uniqueness. Comparative genomics analysis further confirmed the distinct characteristics of strain G01 as an independent lineage. Compared with strains that are phylogenetically closer to it, such as 107105 and ATCC_21228, G01 possesses three unique gene clusters and 221 genes that occur only in this strain. Among these specific genes are those that encode the putative ultraviolet damage repair protein UvrX, a subunit of the antifungal lipopeptide plipastatin synthase, and several genes responsible for the synthesis of spore coat proteins. These genetic elements may endow G01 with increased tolerance to environmental stresses, potential antifungal properties, and a stable spore structure. Together, these characteristics constitute potential advantages in adapting to complex agricultural and pastoral environments.

However, it is essential to clarify that the associations between the genomic traits and phenotypes identified in this study are largely based on the results of sequence analysis and in vitro experiments. We are fully aware of the different levels of evidence at play here. First, the broad-spectrum antibacterial activity of G01, its ability to efficiently degrade proteins and cellulose, and its strong ability to form biofilms are phenotypic traits that have been directly confirmed through experiments. The presence of corresponding gene families with known functions in its genome provides a molecular explanation for these traits. Second, on the basis of known biological mechanisms, we can reasonably infer that its ability to inhibit specific pathogenic bacteria is likely mediated by the identified gene clusters related to antibacterial metabolites; its environmental adaptability and colonization potential may be associated with quorum sensing systems, specific stress response genes, and the predicted secreted proteome. Nevertheless, many of the specific molecular mechanisms remain hypothetical and require further experimental verification.

In summary, the value of *B. subtilis* G01 lies in its combination of biological control potential against specific pathogens, prebiotic functions that enhance digestion, and strong genetic basis for environmental adaptation and colonization, as revealed by genomic and comparative genomics studies. This multifunctional combination of phenotypic and genotypic traits endows it with unique potential for cross-application in sustainable agriculture and animal husbandry. Through multilevel analyses, this study systematically identified the key characteristics of G01 and established a framework for correlating genetic factors with phenotypic traits, thus providing clear objectives and validation pathways for further in-depth mechanistic research and application-oriented development. Future research should focus on conducting detailed functional experiments to verify these conclusions and hypotheses.

## Supplemental Materials

Supplementary data for this paper are available on-line only at http://jmb.or.kr.



## Figures and Tables

**Table 1:** General features of the *B. subtilis* G01 genome.

Features	Chromosome	Plasmid
Genome topology	Circular	Circular
Size (bp)	4,109,649	64,615
G+C content (%)	43.40	38.00
Number of genes	4285	87
tRNA	88	0
rRNA	30	0
CRISPR number	9	2
Prophage	3	0
Genomic islands	19	1
GenBank accession	CP174155	CP174156

**Table 2:** Gene clusters involved in the synthesis of biocontrol metabolites in *B. subtilis* G01.

Gene cluster	Position	Size	Cluster type^a^	Metabolite	Bioactive spectrum^b^
GE000097-GE000139	86,482 − 127,579	41,098 bp	T3PKS	Unknown	Unknown
GE000206-GE000225	176,926 − 198,476	21,551 bp	Terpene	Unknown	Unknown
GE000291-GE000326	247,547 − 296,621	49,075 bp	NRPS, betalactone	Fengycin	Fungi^1^
GE001156-GE001177	1,040,165 − 1,060,971	20,807 bp	Terpene	Unknown	Unknown
GE001922-GE001968	1,797,595 − 1,860,391	62,797 bp	NRPS	Surfactin	Fungi^1^
GE002434-GE002449	2,333,243 − 2,347,789	14,547 bp	RiPP-like	Unknown	Unknown
GE002661-GE002700	2,557,171 − 2,598,589	41,419 bp	Other	Bacilysin	Bacteria^1^
GE002705-GE002723	2,601,898 − 2,623,509	21,612 bp	Sactipeptide	subtilosin A	Bacteria^2^
GE003401-GE003438	3,253,626 − 3,300,762	47,137 bp	NRPS	Bacillibactin	Microbial competitors^3^

^a^Type III polyketide synthase, T3PKS; nonribosomal peptide synthetase, NRPS; other unspecified ribosomally synthesized and posttranslationally modified peptide product, RiPP-like.

^b^1, gene clusters from *Bacillus velezensis* FZB42; 2, gene cluster from *Bacillus subtilis* subsp. *spizizenii* ATCC 6633; 3, gene cluster from *Bacillus subtilis* subsp. *subtilis str*. 168.

**Table 3:** Antimicrobial activity of *B. subtilis* G01.

Gram reaction and strains	Broth medium	Antimicrobial activity^a^
Fungi		
*Candida albicans* ATCC 10231	YM	8.7 ± 0.2 (+)
*Aspergillus niger* ATCC 16404	YM	7.1 ± 0.3 (+)
Gram-positive bacteria		
*Staphylococcus aureus* ATCC 6538	LB	7.2 ± 0.2 (+)
*Micrococcus luteus* ATCC 10240	LB	7.9 ± 0.5 (+)
Gram-negative bacteria		
*Escherichia coli* ATCC 25922	LB	-
*Pseudomonas aeruginosa* ATCC 27853	LB	-
*Listeria monocytogenes* ATCC 19115	BHI	-
*Ralstonia solanacearum* GMI1000	CPG	32.0 ± 1.6 (+++)
*Legionella pneumophila* ATCC 33152	AYE	45.8 ± 2.4 (+++)

^a^The diameter of the inhibition zones was measured in millimeters, and the plus sign indicates the size of the inhibition zones. (+): the diameter of the inhibition zones was less than 10 mm (the size of the wells was 5 mm); (++): the diameter of the inhibition zones was between 10 mm and 15 mm; (+++): the diameter of the inhibition zones was greater than 15 mm. Each experiment was repeated at least three times, and the mean values were used.

**Table 4:** Probiotic properties of *B. subtilis* G01.

Biological function	Activity
Enzyme ability^a^	
Protease	14.3 ± 0.4 (++)
Cellulase	12.6 ± 0.2 (++)
Colonization ability^b^	
Biofilm formation	0.26 ± 0.06 (+)

^a^The diameter of the degradation zones (enzyme ability) was measured in millimeters, and the plus sign indicates the size of the inhibition zones. (+): the diameter of the inhibition zones was less than 10 mm (the size of the wells was 5 mm); (++): the diameter of the inhibition zones was between 10 mm and 15 mm; (+++): the diameter of the inhibition zones was greater than 15 mm.

^b^According to the critical ODc (0.13 ± 0.08) value (ODc is equal to the average value of the blank well plus 3 times the standard deviation), the biofilm can be classified as follows: OD ≤ ODc means no adhesion (−), ODc < OD ≤ 2 ODc means weak adhesion (+), 2 ODc < OD ≤ 4 ODc means medium adhesion (++), and OD > 4 ODc means strong adhesion (+++). Each experiment was repeated at least three times, and the mean values were used.

**Table 5:** Substitution rates of orthologous genes in G01 compared with those in 107105 and ATCC_21228.

Gene	Ka_Ks		Protein	Description
	G01/107105	G01/ATCC_21228		
GE002892	0.69	0.69	Hypothetical protein BSNT_10205	Unknown
GE000408	0.39	0.39	Hypothetical protein	Unknown
GE003056	0.39	0.39	Hypothetical protein	Unknown
GE001944	0.37	0.33	Surfactin nonribosomal peptide synthetase SrfAA	Synthesis of surfactant
GE003207	0.35	0.35	Hypothetical protein	Unknown
GE001943	0.33	0.33	Surfactin nonribosomal peptide synthetase SrfAB	Synthesis of surfactant
GE003420	0.31	0.31	Nonribosomal peptide synthetase DhbF	Iron transporter
