# Supplementary material for: Bacillus subtilis G01: A Multifunctional Agent with Broad-Spectrum Antimicrobial Activity and Digestive Enzymes for Sustainable Agriculture and Animal Husbandry
Source: J Microbiol Biotechnol. 2026 Mar 25;36:e2510049. doi: 10.4014/jmb.2510.10049 (PMC13036507; doi:10.4014/jmb.2510.10049)
Supplement: Supplementary file 1 [file jmb-36-e2510049-supple.pdf]

J. Microbiol. Biotechnol. 2026. 36: e2510049
https://doi.org/10.4014/jmb.2510.10049

Supplementary Figures and Tables

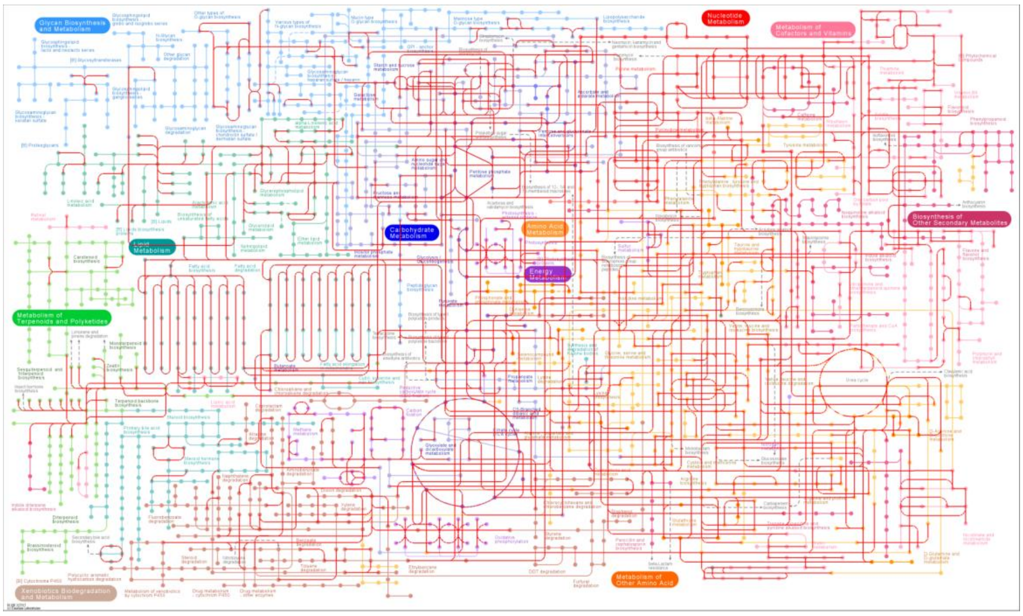

Fig. S1. Global KEGG metabolic map of *B. subtilis* G01.

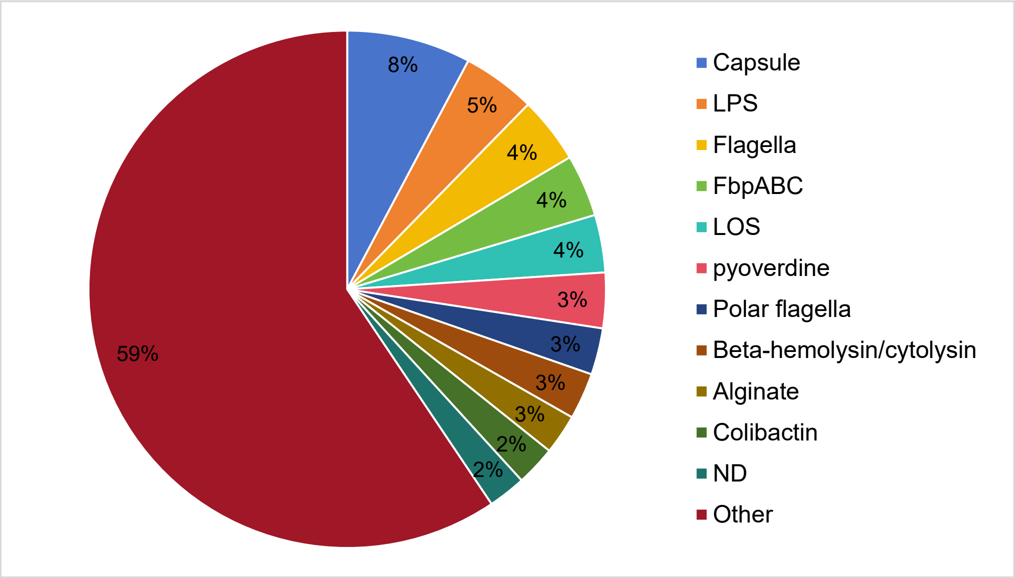

Fig. S2. Virulence factor (VFDB) distribution scale map.

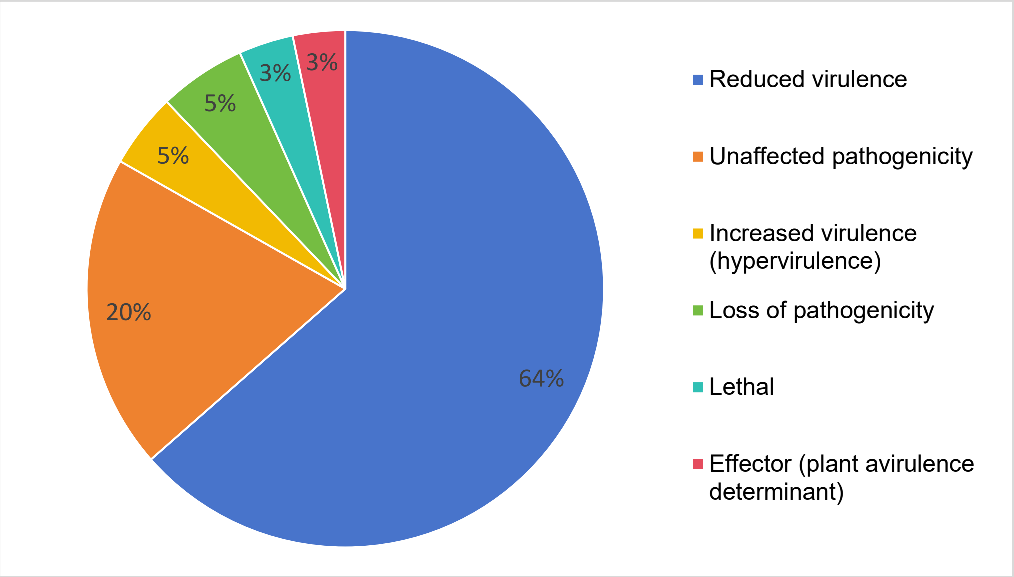

Fig. S3. Pathogen–host interaction (PHI-base) distribution scale map.

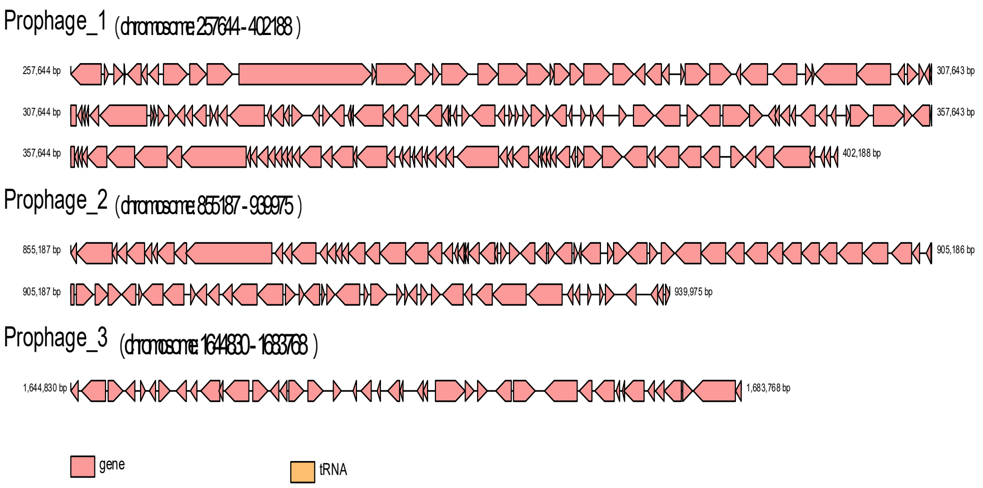

Fig. S4. Prophage regions identified in *B. subtilis* G01.

A

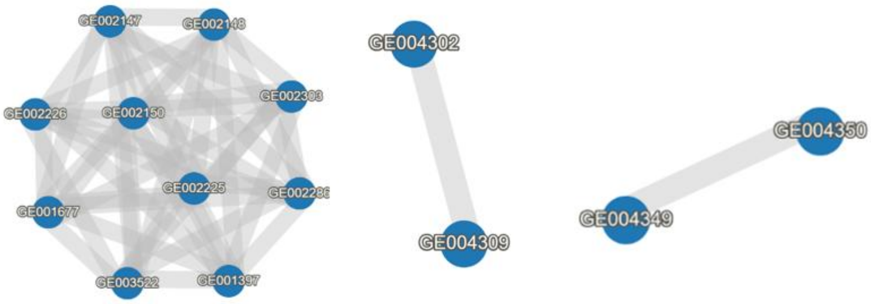

B

Cluster name	Protein number	GO annotation
Cluster8	10	N/A
Cluster4011	2	N/A
Cluster4012	2	GO:0016021; C:integral component of membrane

Fig. S5. ***B. subtilis*** **G01 specific orthologous gene clusters.**

Table S1. The functional genes of plasmid pG01.

Gene	Position	Protein	Description
GE004286	1 - 627	VanZ family protein	Confers low-level resistance to teicoplanin
GE004289	3,072 - 4,337	UV damage repair protein UvrX	Resistant to UV damage
GE004310	14,633 - 15,475	DUF1738 domain-containing protein	N-methyltransferase activity
GE004313	16,679 - 17,206	Thermonuclease	Catalyzed hydrolysis of phosphodiester bond
GE004316	19,496 - 20,308	ATP-dependent DNA ligase	Seals double-strand gaps during DNA repair
GE004318	20,961 - 22,004	DUF4062 domain-containing protein	Uncharacterized protein
GE004322	23,604 - 24,173	Site-specific integrase	DNA binding
GE004325	25,099 - 25,569	Single-stranded DNA-binding protein	May be involved in transcription regulation of the alpha 2(I) collagen gene
GE004327	26,025 - 26,423	Repressor Rok	Repressor of comK
GE004331	28,447 - 29,826	DUF3991 domain-containing protein	metallopeptidase activity
GE004337	33,662 - 34,768	Lipoprotein	integral component of membrane
GE004343	41,122 - 43,482	VirD4 component of type IV secretory pathway	integral component of membrane
GE004354	49,345 - 50,553	CpaF family protein	ATP binding
GE004361	55,119 - 55,403	Phage protein	Mediates plasmid DNA transduction
GE004365	58,424 - 58,909	XRE family transcriptional regulator	sequence-specific DNA binding
GE004367	59,211 - 60,317	Response regulator aspartate phosphatase C	Inhibits the activity of ComA
GE004368	61,703 - 62,878	Actin-like protein Alp7A	Involved in plasmid separation

Note: The plasmid pG01 includes 87 CDS consisting of the gene id from GE004286 to GE004372. seventeen genes with functional annotation are list in the table, while the functions of other genes are unknown.

Table S2. Antibiotic resistance genes identified in *B. subtilis* G01.

Gene ID	Position	Resistance type	Description
GE000900	823,699 - 824,016	ykkd	ykkd is an SMR-type protein that is a subunit of the ykkCD efflux pump
GE000901	824,016 - 824,354	ykkc	ykkC is an SMR-type protein that is a subunit of the ykkCD efflux pump
GE001431	1,313,30 - 1,315,879	mprF	MprF is a integral membrane protein that modifies the negatively-charged phosphatidylglycerol on the membrane surface. This confers resistance to cationic peptides that disrupt the cell membrane, including defensins. Additionally, large-scale mutations causing loss of function of the gene result in increased susceptibility to daptomycin.
GE001709	1,610,635 - 1,612,281	vmlR	VmlR is an ABC-F ATPase ribosomal protection protein identified in Bacillus subtilus. Shown to confer resistance to lincomycin and streptogramin A virginiamycin.
GE001988	1,882,387 - 1,882,980	tmrB	tmrB is an ATP-binding tunicamycin resistance protein found in Bacillus subtilis.
GE002040	1,930,598 - 1,932,031	lmrB	lmrB is a chromosomally-encoded efflux pump that confers resistance to lincosamides in Bacillus subtilis.
GE002055	1,943,926 - 1,944,846	mphK	A chromosomal macrolide phosphotransferase identified from Bacillus subtilis.
GE002870	2,754,333 - 2,755,475	pgsA with mutation conferring resistance to daptomycin	Point mutations that occur within the Bacillus subtilis pgsA gene resulting in resistance to daptomycin.
GE003950	3,825,235 - 3,826,089	aadK	aadK is a chromosomal-encoded aminoglycoside nucleotidyltransferase gene in B. subtilis and Bacillus spp.
GE003970	3,841,984 - 3,843,186	blt	blt is an MFS efflux pump that confers resistance to multiple drugs such as rhodamine and acridine dyes, and fluoroquinolone antibiotics.
GE004179	4,024,840 - 4,025,907	bmr	bmr is an MFS antibiotic efflux pump that confers resistance to multiple drugs including acridine dyes, fluoroquinolone antibiotics, chloramphenicol, and puromycin.

Table S3. Representative genes of *B. subtilis* G01 probably involved in host-bacteria interactions.

#	Gene	Position	Protein description
**Putative secreted proteins**	GE000203	174,010 - 175,254	peptidoglycan endopeptidase
	GE000718	652,827 - 653,456	YhcN/YlaJ family sporulation lipoprotein
	GE000826	755,289 - 755,915	cell wall hydrolase
	GE000915	835,072 - 836,703	peptide ABC transporter substrate-binding protein
	GE001307	1,194,059 - 1,195,063	peptidoglycan endopeptidase
	GE001312	1,200,802 - 1,202,262	peptidoglycan endopeptidase
	GE001333	1,224,436 - 1,225,011	YhcN/YlaJ family sporulation lipoprotein
	GE001517	1,404,259 - 1,405,521	polysaccharide lyase family 1 protein
	GE001570	1,462,128 - 1,463,411	carbohydrate ABC transporter substrate-binding protein
	GE001602	1,498,157 - 1,499,347	CamS family sex pheromone protein
	GE001873	1,752,228 - 1,754,234	penicillin-binding protein 3
	GE002001	1,895,489 - 1,896,370	glycine/betaine ABC transporter substrate-binding protein
	GE002136	2,026,376 - 2,027,701	penicillin binding protein PBP4B
	GE002153	2,056,876 - 2,057,433	spore germination protein GerD
	GE002567	2,464,422 - 2,465,261	polysaccharide deacetylase family protein
	GE002976	2,866,199 - 2,866,864	pectate lyase
	GE003074	2,953,811 - 2,955,232	glycoside hydrolase family 68 protein
	GE003147	3,028,665 - 3,029,585	osmoprotectant ABC transporter substrate-binding protein
	GE003775	3,648,147 - 3,649,247	germination protein GerM
	GE004001	3,867,218 - 3,867,628	sporulation-specific extracellular nuclease
	GE004240	4,075,838 - 4,077,007	D-alanyl-D-alanine carboxypeptidase
**Quorum sensing**	GE003443	3,304,238 - 3,305,185	quorum-sensing protein
	GE003444	3,305,187 - 3,305,408	competence pheromone ComX

GE001581	1,471,666 - 1,471,839		phosphatase RapH inhibitor PhrH
GE001914	1,789,254 - 1,789,376		Secreted regulator of the activity of phosphatase RapC (CSF)
GE004157	4,001,732 - 4,002,535		sporulation transcription factor Spo0A
GE002386	2,286,643 - 2,286,759		Phr family secreted Rap phosphatase inhibitor

Table S4. Results of the whole genome sequence comparison.

Strain	Genome size (bp)	GC content(%)	Protein -coding gene	tRNA number	rRNA number	Isolation source	Accession number	Country
G01	4,174,264	43.40	4,372	88	30	air environment	CP174155	China
107105	4,189,350	43.50	4,149	87	30	natto	CP121266	China
ATCC_21228	4,226,648	43.50	4,161	87	30	soybeans	CP020023	USA

Table S5. Representative singletons genes of *B. subtilis* G01.

**Gene**	**Position**	**Protein description**
GE000156	140,327 - 140,638	spore maturation protein CgeA
GE000255	220,201 - 220,482	Putative UV-damage repair protein UvrX
GE000256	220,448 - 220,657	Putative UV-damage repair protein UvrX
GE000308	275,387 - 277,654	Plipastatin synthase subunit
GE000388	344,206 - 345,360	IS256 family transposase
GE000559	495,561 - 496,244	flagellar type III secretion system protein FliR
GE001747	1,643,901 - 1,644,122	HxlR family transcriptional regulator
GE001993	1,887,113 - 1,888,333	lactate permease
GE002358	2,257,433 - 2,257,552	spore coat protein CotF
GE003915	3,792,487 - 3,793,824	peptidoglycan O-acetyltransferase
GE003983	3,853,598 - 3,853,810	Antitoxin YqcF

Table S6. Comparison of *B. subtilis* G01 with other *B. subtilis* strains.

	Source	CAZyme	Secondary Metabolism Gene Clusters	Antimicrobial Spectrum	Enzymatic Activity	Core Features	References
G01	Isolation from air	Primarily GH and GT	Fengycin, Surfactin, Bacilysin, Subtilosin A, Bacillibactin and 4 unannotated	Strongly inhibits plant pathogen and zoonotic pathogen, but has no inhibitory effect on common intestinal bacteria.	Protease + cellulase synergistic high efficiency	Dual activity of biological control and probiotics	/
BS21	Isolation from the feces of pigs	/	Surfactin, Zwittermicin A, Bacillaene, Fengycin, Bacillibactin, Subtilosin A, Bacilysin and 6 unannotated	Primarily inhibits pathogenic bacteria in animals	/	As an antibiotic alternative probiotic, it maintains the balance of animal gut microbiota and is suitable for animal husbandry	[1]
Bbv57	Isolation from the rhizosphere of a betelvine	Primarily GH and GT	Bacillibactin, Bacilysin, Subtilosin A, Fengycin, Piplastin, Paenibactin, Surfactin and 2 unannotated	Inhibits fungi, bacteria, and nematodes, demonstrating significant antagonistic effects against plant soil-borne pathogens	Antibiotic resistance-related enzymes	Plant biological control, inhibiting the infection of plant pathogenic bacteria and nematodes	[2]
KC14-1	Isolation from soil samples	Primarily GH and GT	Surfactin, Fengycin, 1-carbapen-2-em-3-carboxylic acid, Bacillibactin, Pulcherriminic acid, Subtilosin A, Bacilysin and 2 unannotated	Broad-spectrum antifungal	Cell wall degradation-related enzymes	Plant biological control, broad-spectrum antifungal activity	[3]
MC4-2	Isolation from the *Periplaneta americana* intestine	Primarily GH and GT	Surfactin, Bacillaene, Fengycin, Bacillibactin, Subtilosin A, Bacilysin and 4 unannotated	Broad-spectrum antifungal/antiviral, core control for Tobacco Black Shank Disease	Antibiotic-related enzymes, metabolic and modification-related enzymes	As a plant biological control agent, it exhibits significant antagonistic effects against tobacco black shank	[4]
DG101	Isolation from Japanese natto	/	/	Broad-spectrum inhibition of Gram-positive/negative bacteria and fungi	Collaborative action of multiple enzymes: amylase, cellulase, lipase, protease, phytase and nattokinase	Probiotics, Health Benefits, Bio-Repair and Antibacterial Protection	[5]

References

[1] WU D, FU L, CAO Y, et al. Genomic insights into antimicrobial potential and optimization of fermentation conditions of pig-derived Bacillus subtilis BS21 [J]. Front Microbiol, 2023, 14: 1239837.

[2] THIRUVENGADAM R, GANDHI K, VAITHIYANATHAN S, et al. Complete Genome Sequence Analysis of Bacillus subtilis Bbv57, a Promising Biocontrol Agent against Phytopathogens [J]. Int J Mol Sci, 2022, 23(17).

[3] LI X, CHEN Y, YANG S, et al. Whole genome-sequence analysis of Bacillus subtilis strain KC14-1 with broad-spectrum antifungal activity [J]. BMC Genomics, 2025, 26(1): 319.

[4] SHI C, ZENG S, GAO X, et al. Complete Genome Sequence Analysis of Bacillus subtilis MC4-2 Strain That against Tobacco Black Shank Disease [J]. Int J Genomics, 2024, 2024: 8846747.

[5] LEñINI C, RODRIGUEZ AYALA F, GOñI A J, et al. Probiotic properties of Bacillus subtilis DG101 isolated from the traditional Japanese fermented food nattō [J]. Front Microbiol, 2023, 14: 1253480.
